# Metagenomic analysis reveals Hepatitis A virus in suspected yellow fever cases in Brazil

**DOI:** 10.1590/0074-02760170260

**Published:** 2018-01

**Authors:** Liliane C Conteville, Ana Maria B de Filippis, Rita Maria R Nogueira, Marcos César L de Mendonça, Ana Carolina P Vicente

**Affiliations:** 1 Fundação Oswaldo Cruz-Fiocruz, Instituto Oswaldo Cruz, Laboratório de Genética Molecular de Microrganismos, Rio de Janeiro, RJ, Brasil; 2 Fundação Oswaldo Cruz-Fiocruz, Instituto Oswaldo Cruz, Laboratório de Flavivírus, Rio de Janeiro, RJ, Brasil

**Keywords:** hepatitis A virus, yellow fever virus, metagenomics, Brazil

## Abstract

Using a metagenomic approach, we identified hepatitis A virus among cases of acute febrile illnesses that occurred in 2008-2012 in Brazil suspected as yellow fever. These findings reinforce the challenge facing routine clinical diagnosis in complex epidemiological scenarios.

In tropical countries, acute febrile illnesses (AFIs) have a broad spectrum of possible aetiologies. In Brazil, Dengue virus (DENV), Chikungunya virus (CHIKV), Zika virus (ZIKV), and Yellow fever virus (YFV) are currently the major arbovirus infections associated with AFIs. DENV became endemic following its re-emergence in 1986, and CHIKV and ZIKV were introduced in the region in the last three years (Figueiredo 2015). More recently, YFV showed an upsurge, representing the most severe YFV epidemic in the country in the last 50 years (Bonaldo et al. 2017). Given such a complex situation, establishment of an AFI laboratory-confirmed diagnosis is fundamental to determine the accurate epidemiological scenario as well as initiate effective and timely control measures and treatment. However, misidentifications are quite common due to the high cross-reactivity between flaviviruses and co-infecting viruses (Felix et al. 2017). Moreover, negative cases represent an additional threat to public health, since they can be due to unsuspected infectious agents with the potential to disseminate silently, as was the case in the early stage of the recent ZIKV epidemic in Brazil (Campos et al. 2015).

In this study, we applied a metagenomic approach to elucidate the prevalence of AFI cases occurring in Brazil in 2008-2012 that were suspected to be YF given that the patients had travelled to sylvatic YFV endemic regions. During the 20th century, YFV was endemic to the northern and central-western Brazilian regions. However, between 2007 and 2009, the YFV circulation area expanded and reached the southeastern and southern regions. Since 2016, YFV has been causing outbreaks that affect non-human primates and unvaccinated human populations from rural areas of southeastern Brazilian states (Minas Gerais, Espírito Santo, Rio de Janeiro and São Paulo) (Mir et al. 2017).

The cases studied herein were all deemed to be negative for the major arboviruses (DENV and YFV) occurring in Minas Gerais in 2012 based on serological and specific reverse transcription-polymerase chain reaction (RT-PCR) analyses. To determine the presence of infectious agents in these cases, we applied metagenomics as reported previously (Conteville et al. 2015). The DNA and RNA from five serum samples were amplified by random RT-PCR, mixed as a pool of samples, and sequenced on an Illumina HiSeq 2500 platform system (Oswaldo Cruz Foundation, high-throughput sequencing platform) using 2 × 100-bp paired-end reads generated with Nextera XT libraries. The metagenomic sequences were analysed with reference to the Kraken (Wood & Salzberg 2014) standard database that includes bacteria, archaea, and viral genomes.

Surprisingly, the analysis resulted in the identification of only one infectious agent: hepatitis A virus (HAV). The reads assigned as HAV comprised 941 bp of the reference genome (EU526088) encompassing the following partial genomic regions: the 5′ untranslated region (UTR), VP3, VP1, 2A, 2C, and 3D. To confirm this result, a specific PCR assay targeting the HAV 5′ UTR (Endo et al. 2007) was performed, and Sanger sequencing of the amplicons confirmed the presence of HAV in two of the five samples. Phylogenetic analysis of the HAV 5′ UTR sequences (403 bp) further revealed that the HAV isolated from both cases belong to the sub-genotype 1A (Figure), which is the most prevalent genotype in Brazil and other South American countries (Fernández et al. 2012). Both patients were residents of Rio de Janeiro and presented the following symptoms after returning from Caxambu, Minas Gerais: fever, nausea, and abdominal discomfort. One of the patients also presented thrombocytopenia, leucopoenia, and hepatosplenomegaly, while the other patient also presented retro-orbital pain and myalgia.

**Figure:**
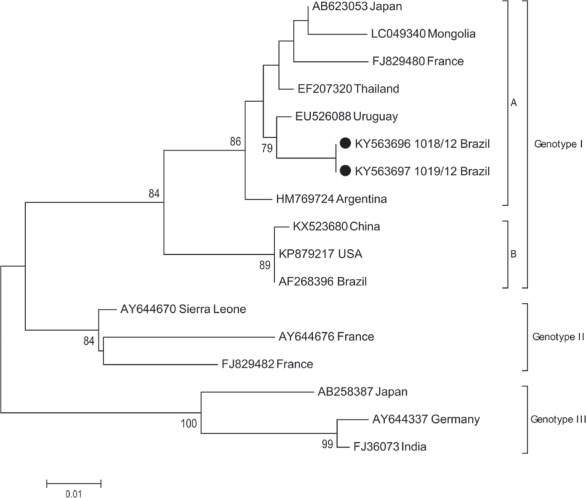
Maximum-likelihood phylogenetic tree showing three genotypes of hepatitis A virus based on the 5′ UTR sequence (403 bp). Sequences derived from this study are labelled in black circles. Numbers beside internal branches indicate bootstrap values based on 500 replicates. The scale bar indicates base substitutions per site.

HAV infection has long been endemic in Brazil, and in 2012, when the two cases from the present study occurred, 600 cases were reported in the country (MS 2015). Viral hepatitis might lead to severe liver failure; however, acute hepatitis A is generally self-limited, and in complex epidemiological scenarios, with the co-occurrence of arboviruses, this infection would not be the main suspicion. Therefore, most HAV infections may be not investigated, leading to underestimation of the prevalence of infections caused by this virus. In fact, ongoing outbreaks of HAV are currently impacting European countries. Interestingly, these outbreaks are attributed to HAV genotype 1A (Werber et al. 2016), which is the same genotype identified in the present Brazilian cases. AFIs can be associated with several types of infections, including those caused by viruses, bacteria, and parasites, and each type of infection requires distinct treatment and control measures. Therefore, this study reinforces the importance of establishing unbiased methods for diagnosis considering the challenges of routine clinical practice, particularly when new complex epidemiological scenarios occur.
